# Comparative Investigation of Activated Carbon Electrode and a Novel Activated Carbon/Graphene Oxide Composite Electrode for an Enhanced Capacitive Deionization

**DOI:** 10.3390/ma13225185

**Published:** 2020-11-17

**Authors:** Gbenro Folaranmi, Mikhael Bechelany, Philippe Sistat, Marc Cretin, Francois Zaviska

**Affiliations:** Institut Européen des Membranes, IEM, UMR-5635, University of Montpellier, ENSCM, CNRS, Place Eugène Bataillon, CEDEX 5, 34095 Montpellier, France; gbenro.folaranmi@etu.umontpellier.fr (G.F.); philippe.sistat@umontpellier.fr (P.S.); marc.cretin@umontpellier.fr (M.C.)

**Keywords:** electro-sorption, electrode, activated carbon, graphene oxide, cyclic voltammetry

## Abstract

Capacitive deionization is an emerging brackish water desalination technology whose principle lies in the utilization of porous electrodes (activated carbon materials) to temporarily store ions. Improving the properties of carbon material used as electrodes have been the focus of recent research, as this is beneficial for overall efficiency of this technology. Herein, we have synthesized a composite of activated carbon/graphene oxide electrodes by using a simple blending process in order to improve the hydrophilic property of activated carbon. Graphene oxide (GO) of different weight ratios was blended with commercial Activated carbon (AC) and out of all the composites, AC/GO-15 (15 wt.% of GO) exhibited the best electrochemical and salt adsorption performance in all operating conditions. The as prepared AC and AC/GO-x (x = 5, 10, 15 and 20 wt.% of GO) were characterized by cyclic voltammetry and their physical properties were also studied. The salt adsorption capacity (SAC) of AC/GO-15 at an operating window of 1.0 V is 5.70 mg/g with an average salt adsorption rate (ASAR) of 0.34 mg/g/min at a 400 mg/L salt initial concentration and has a capacitance of 75 F/g in comparison to AC with 3.74 mg/g of SAC, ASAR of 0.23 mg/g/min and a capacitance of 56 F/g at the same condition. This approach could pave a new way to produce a highly hydrophilic carbon based electrode material in CDI.

## 1. Introduction

Obtaining alternative sources of fresh water has been one of the foremost challenges in this era of constant increases in population. Human developments such as the process of industrialization have led to an upsurge in climatic changes and as a result, the hydrological cycle has been affected. Water shortages have blighted many regions of the world [1] (causing adverse physical and economic impacts) and there is an urgent necessity to circumvent this issue by developing a technology that can augment the existing water desalination technologies by exploiting surface water with a low salt concentration (brackish water).

Capacitive Deionization (CDI) is a water desalination technology that has been engineered for highly efficient functionality when desalting brackish water [2]. CDI makes use of porous carbonaceous materials as solid electrodes, hence making it a very cheap process in comparison to other existing water technologies like reverse osmosis, membrane distillation [3], etc. The basic principle of CDI lies in the storage of ions (adsorption) when an external direct voltage (usually below 2.0 V) is applied to a system containing a brine solution; the electrically induced charged ions in the solution are adsorbed into the surface of the polarized electrodes (electrical double layer interface) [4] and as soon as the external electric field is reversed, the adsorbed ions are desorbed and fed back to a bulk solution, leading to a consequent regeneration of saturated electrode materials.

The materials considered for CDI electrodes are commonly selected based on possessing properties or characteristics such as high hydrophilicity, high porosity, high surface area, good electronic conductivity, etc. and since activated carbon (AC) fits into some of these criteria, it is therefore used for CDI electrodes. However, because there are some downsides to using AC alone, i.e. low pore accessibility though high surface area, low conductivity, low hydrophilicity etc. hence, it is usually used in a combined or composite state in order to compensate for its shortcomings [2]. AC has mainly found applications in environmental studies [5,6,7]; herein it is instead converted into electrodes and modified for CDI applications.

One of the major factors governing the performance of the CDI process is the electrode materials used for the process [8,9], therefore a great deal of effort is focused on improving these materials. Research in this area has focused on improvements in terms of carbon precursors, composite formation (hybridization), surface chemistry, etc.

Hybrid composites of activated carbon offer higher performance outcomes, as has been reported in the literature [10,11,12,13]. In this paper, we have successfully combined the properties of AC and graphene oxide (GO) to form hybrid composites in order to improve electrode wettability and consequently studied the electrochemical and electrosorption effects of this process. We note here that there are limited reports focusing on this area, as most wettability processes involving CDI focus on carbon oxidation.

GO has been used for improving the hydrophilicity of many materials such as a zeolitic imidazole framework [10], membrane bioreactor [14] and cotton fabrics [15], but this application of GO in CDI can be scarcely found in the literature [16], as most research in this area is focused on graphene applications for the electronic improvement of AC [17,18,19].

Motivated by this, we have prepared a highly hydrophilic activated carbon doped graphene oxide by using a simple blending process to improve the wettability of AC. To reach the best compromise, activated carbon doped graphene oxides of different compositions were synthesized and labeled as AC/GO-x, where x represents the weight percentage of GO in the composite (x = 5, 10, 15 and 20). The electrochemical properties of the prepared electrodes (AC, AC/GO-x) were analyzed by using cyclic voltammetry; afterwards, the most efficient electrode was tested in comparison with pristine AC in the constructed CDI cell.

## 2. Materials and Methods

### 2.1. Materials

Polyvinylidenefluoride (PVDF) (CAS no: 24937-79-9, Alfa Aesar, Erlenbachweg 2, Kendel, Germany), *N*-Methyl-2-pyrrolidone (NMP) (CAS no 872-50-4, 99.7%, M.W 99.13 g/mol), Hydrochloric acid (HCl) (CAS no: 7647-01-0, 37%), Activated carbon (Supelco Analytical, CAS no: 7440-44-0) and graphite powder (CAS no: 7782-42-5) were supplied by Sigma Aldrich, Steinheim, Germany. Carbon black (CAS no: 1333-86-4) was supplied by Alfa Aesar (Steinheim, Germany). Graphite foil (0.35 mm thick) was supplied by RMC Remacon, Bad Säckingen, Germany.

Phosphoric acid (H_3_PO_4_), (CAS no: 7664-38-2, 85%) and potassium permanganate (KMnO_4_) (CAS no: 7722-64-7, 98%) were purchased from Alfa Aesar (Kandel, Germany). Sulfuric acid (H_2_SO_4_) was purchased from ACS Reagent, Belmont, CO, USA. Absolute ethanol (CAS no: 64-17-5, 99.8%) and hydrogen peroxide (CAS no 7722-84-1, 7732-18-5 30%) were purchased from VWR chemicals, Paris, France. All chemicals were used as supplied.

### 2.2. Experimental Procedure

#### 2.2.1. GO Synthesis

GO was synthesized using Marcano’s method [20]. A total of 5 g of graphite powder and 18 g of potassium permanganate was slowly added to a mixture of 40 mL of phosphoric acid and 360 mL of sulfuric acid. The solution was stirred for 18 h at room temperature, then 3 mL of H_2_O_2_ was added. The solution was then filtered and centrifuged (4000 rpm for 10 min) so the supernatant could be decanted away. The mixture was then washed multiple times with 30% HCl and distilled water, following the procedure described elsewhere [13]. The obtained GO was placed in an oven for 4 h at 80 °C.

#### 2.2.2. Fabrication of Activated Carbon/AC/GO-x Electrodes

Activated carbon electrode was prepared using commercial activated carbon powder of high specific surface area as determined by N_2_ adsorption (1034 m^2^/g). Carbon slurry was prepared as a suspension of activated carbon powder (3.2 g), carbon black (0.4 g) and poly(vinylidene fluoride PVDF, 0.4 g) in 25 mL *N*-Methyl-2-pyrrolidone (NMP). The mixture was stirred for 2 h and sonicated for 40 min to ensure homogeneity. The slurry was then deposited on a graphite sheet by using spray coating (Air brush Iwata, Fukushima, Japan). It was spray coated onto a graphite sheet with a thickness of 0.357 mm. The coated electrode was dried at 80 °C in an oven for 3–5 h. For the composite synthesis, the slurry was prepared by adding the as-prepared GO (5, 10 15 and 20 wt.%) to AC and the aforementioned procedure (for carbon slurry preparation) was followed.

### 2.3. Physical Characterization

Field emission scanning electron microscopy (FESEM) was used to analyze the structure of the samples (FESEM, Hitachi S4800, Tokyo, Japan). The structural properties were studied by using Raman spectroscopy (HORIBA Xplora, Minami-ku Kyoto, Japan). Water contact angle (WCA) testing was performed to understand the nature of surface interaction of the materials when in contact with water, and X-ray diffractometer (XRD Pan Analytical X’pert Phillips, Lelyweg, The Netherlands) revealed the crystallinity of the materials. X-ray photon electron spectroscopy (XPS) (ESCALAB 250 Thermo Electron, Strasbourg, France) was done to investigate the atomic composition and chemical functional groups of the materials. For the XPS analysis, the excitation source was a monochromatic source Al Kα anode with photo energy that was observed at 1486.6 eV. The analyzed surface has a diameter of 500 µm. The photoelectron spectra were calibrated in terms of bond energy with respect to the energy of the C=C component of carbon C1s at 284.4 eV. Surface area was obtained by using N_2_ adsorption/desorption at 77 K. S_BET_ was the specific surface area calculated by the Brunauer-Emmett-Teller (BET) method (Micromeritics 2020 ASAP, Merignac, France). Vt was the total pore volume calculated from the amount adsorbed at a relative pressure (P/P^0^) of 0.99, V_meso_ was the mesopore volume calculated by the Barrett-Joyner-Halenda (BJH) model. The micropore volume was calculated from a T-plot using a constant conversion factor of 0.0015468 multiplied by the corresponding volume of gas adsorbed (cm^3^/g STP) at a relative pressure (P/P^0^) of 0.1.

### 2.4. Electrochemical Characterizations

The electrochemical properties of the as prepared electrodes were examined by using cyclic voltammetry (CV). CV tests were performed using a three-electrode system. The carbon electrode (deposited on a graphite sheet as support) with an exposed surface area of 1 cm^2^ was made to have contact with the electrolyte (0.5 M NaCl solution), while a platinum rod and a saturated Ag/AgCl served as counter and reference electrodes, respectively. Voltammetry measurements were performed with Origalys Potentiostat (OGF01A, Origalys Electrochem SAS, Rillieux-la-Pape, France) at an operating window from −0.4 to 0.6 V vs. ref (0.1 V) in a 0.5 M NaCl electrolyte.

The double-layer capacitance was determined using cyclic voltammetry at different scan rates by considering the open circuit potential (OCP 0.1 V vs. ref) of the cathodic and the anodic currents. The determined double-layer capacitance of the system was the average of the absolute value of the slope of the linear plot of cathodic and anodic regions fitted to the data. C as the specific capacitance (F/g) was then determined considering the mass (g) of the active material on the electrode surface (1 cm^2^) using Equation (1)

i = *v*C_DL_ (1)

For an ideal capacitor Q = CV, thus by differentiation i = C*v*, where *v* is the scan rate.

The double-layer charging current i is equal to the product of the scan rate, *v*, and the electrochemical double-layer capacitance, C_DL_.

### 2.5. CDI Measurements

**Figure 1:**
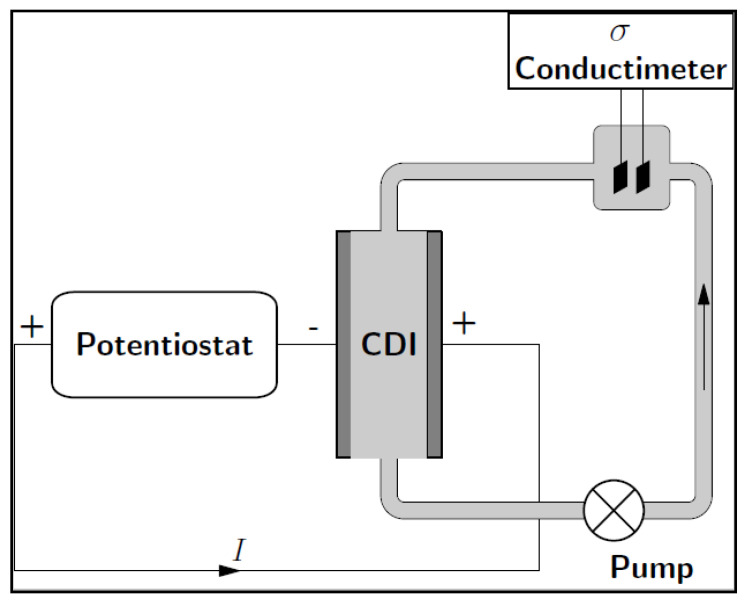
Schematic diagram of a capacitive deionization (CDI) set up.

**Figure 2:**
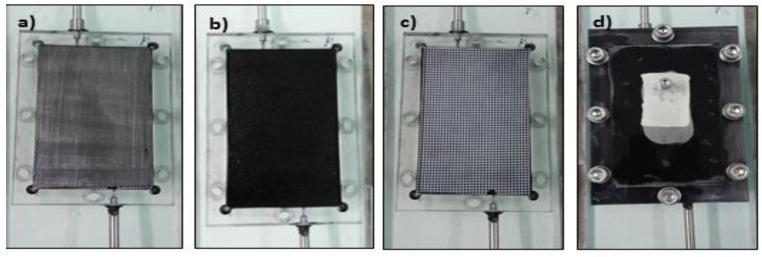
CDI, (**a**) Open cell with a current collector, (**b**) current collector with electrode, (**c**) solid electrode with a separator, (**d**) closed cell.

The CDI performance of the carbon materials was evaluated in a recycling system (batch mode), as shown in Figure 1. It comprised a CDI cell, a peristaltic pump, a direct current power source (Origalys PST, Rillieux-la-Pape, France) and a conductivity meter (Hannah TECH, Cluj-Napoca, Romania). Figure 1 shows the constructed CDI cell; it consisted of the cell containing the two parallel electrode sheets separated by a non-electrically conductive spacer (0.99 mm thick), as displayed in Figure 2. The electrode materials were directly attached to the current collector, which was subsequently connected to an external power source (potentiostat). The CDI electrodes have an area of 6 × 10 cm^2^ and brine solution (400 and 1200 mg/L NaCl) was continually pumped into the CDI cell at a constant rate of 25 mL min^−1^. The conductivity change of the brine solution (feed solution) was monitored at room temperature. The CDI tests were conducted at potential differences of 1.0 V and 1.4 V.

Salt adsorption capacity (SAC) is defined as the adsorbed amounts of ions per gram of electrode and was calculated by the variation in concentration of the brine solution that was being monitored by a conductivity meter. Adsorption capacity Q (mg/g) in CDI was defined by Equation (2):

(2) $$Q\;=\;\frac{\left(C_{i}-C_{f}\right)V}{m}$$

where C_i_ and C_f_ are the initial and final concentration (mg/L), respectively, V is the volume of the solution (L), and m is the total mass of the deposited electrodes (g).

Adsorption efficiency was calculated by Equation (3):

(3) $$\mathrm{Efficiency}\;(\%)\;=\;\frac{C_{i}-C_{f}}{C_{i}}\times 100$$

Charge efficiency is the ratio of salt adsorbed to the quantity of charge passed into the system and was calculated by Equation (4):

(4) $$\mathrm{CE}\;=\;\frac{z\;\left(C_{i}-C_{f}\right)\;V\;F}{⟆Idt}$$

where z is the equivalent charge of the ions, C_i_ and C_f_ are the initial and final concentration (mg/L), respectively, V is the volume of the solution (L), F is the Faradaic constant and ⟆Idt is the integrated quantity of charge passed to the system as a function of time.

## 3. Results

In order to get the best ratio or to reach a compromise for the most efficient composite electrodes, AC and AC/GO-x where x represents the weight percentage of GO in the composite (x = 5, 10, 15 and 20) were prepared and characterized by using both physical and electrochemical means.

### 3.1. Morphological Properties

**Figure 3:**
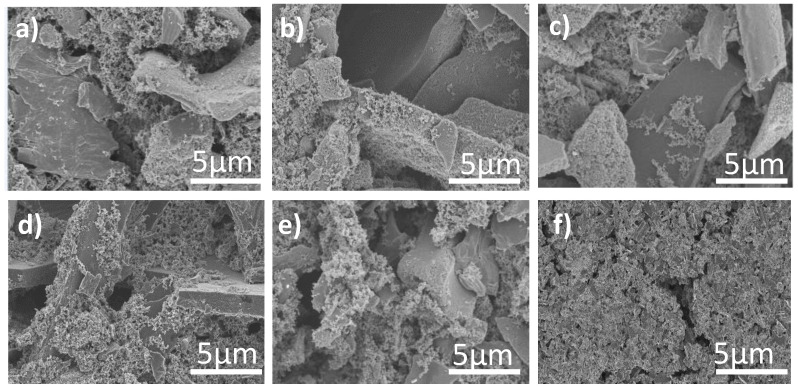
Field emission scanning electron microscope (FESEM) top view of: (**a**) AC (**b**) AC/GO-5 (**c**) AC/GO-10 (**d**) AC/GO-15 (**e**) AC/GO-20 and (**f**) GO-electrode.

SEM was performed to visualize the morphology of the commercial AC and also to verify if the addition of GO at different ratios would have a significant effect on the morphology of AC. As shown in Figure 3a–f, there was no significant change in morphology of the composites (AC/GO-5, AC/GO-10, AC/GO-15 and AC/GO-20) when compared with that of pristine AC electrode probably due to the homogenous dispersion of the GO in the activated carbon. The composites were hardly differentiable from the pristine AC. Both AC and its composites have indefinite shapes with rough surfaces.

### 3.2. Raman Analysis of GO, AC and AC/GO-x

**Figure 4:**
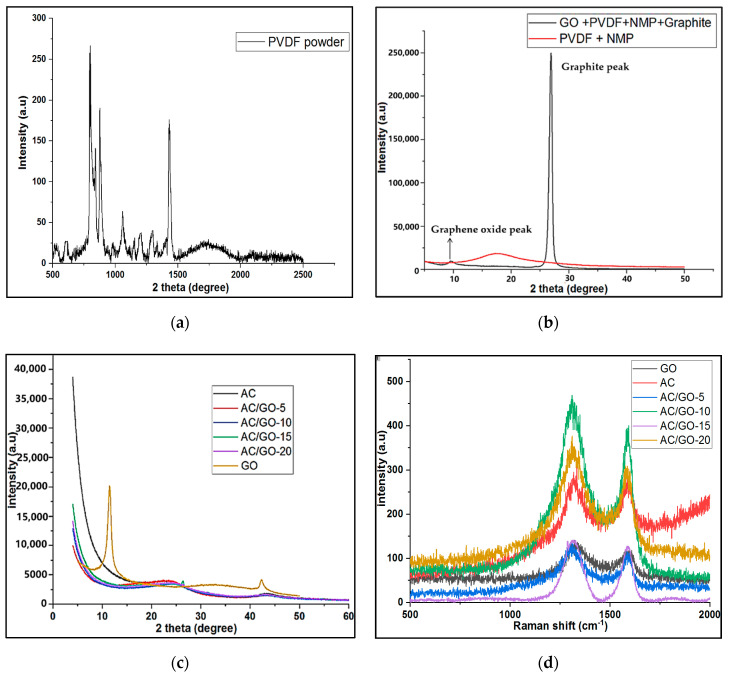
X-ray diffraction (XRD) of (**a**) Pure PVDF powder (**b**) PVDF dispersed in NMP solvent containing GO and graphite. (**c**) AC, GO and AC/GO-x electrodes. Raman spectroscopy of (**d**) AC, GO and AC/GO-x electrodes.

Raman analysis was performed in order to understand the defect that might have occurred on the lattice of pristine AC due to the addition of GO. As shown in Figure 4d, all the electrode materials conformed to the D-band at 1350 cm^−1^ due to the disordered graphite (out of plane vibration) and the G-band at 1580 cm^−1^ due to ordered graphite (in-plane vibration) [21]. Intensity ratio (R = I_D_/I_G_) in Raman gives us information of the level of defect present in any carbonaceous material. The intensity ratio of our pristine AC was 1.04 while that of GO is 1.25 (this intensity is high due to the effect of exfoliation process involved in making GO while that of AC might be due to the destructive distillation process involved in its synthesis from coke). A slight increase of defects was observed in the composite AC/GO-x (x = 5, 10, 15 and 20) with a range of intensity ratio of 1.02–1.17, indicating a low degree of graphitization (a low level of graphitic domain) in all of the materials [22].

### 3.3. XRD Investigation

XRD was used to understand the amorphous or crystalline nature of AC and composite electrodes. For a typical crystalline carbon material, a sharp diffraction peak was observed at 2θ = 25 and 45° corresponding to the 002 and 100/101 planes [23], respectively. As evident in Figure 4a, broad and diffuse diffraction peaks were observed for the as prepared AC and its corresponding composite electrodes, indicating a low degree of crystallinity and graphitic structure of the materials which corroborates with Raman analysis. The crystallinity of the AC in the composite was not enhanced by the addition of crystallized GO, which was made evident with a sharp diffraction peak at 10.0° (typical diffraction peak of a crystalline GO) [23]. No diffraction peak of GO was observed in all the composites due to its high dispersity [13].

The XRD peak of graphite was observed at 2θ = 26.6° while after exfoliation, new peak of GO at 2θ = 10.0° was observed, as shown in Figure 4b. We used Bragg’s law (*n*λ = 2dsinθ), where *n* is 1, λ is X-ray diffraction (1.541 A°), θ is the angle of diffraction in the degree and d is the inter planar distance between graphite layers. The inter-planar distance between graphite and GO was calculated and observed to be 0.3 nm and 0.9 nm, respectively. This shows that there was an increase in the inter-layer spacing in the graphite layer due to the introduction of oxygenated functional groups via chemical oxidation [24]. There was no difference in the inter-layer spacing of pristine AC and its composites, as shown in Table 1. In a typical carbonaceous material, i.e., graphite, the interlayer distance between two adjacent carbon sheets was 0.33 nm as calculated [25] but that of AC and its corresponding composites at 2θ = 25° which corresponds to an interlayer distance (d002) of 0.36 nm suggests a disordered carbonaceous interlayer graphitic material; a result corroborating Raman analysis. Figure 4a shows the XRD pattern of pure PVDF binder but when dispersed in NMP (solvent), it became amorphous and could hardly be detected [21] (see Figure 4b for further details), hence its peak was not observed in the diffractogram of the electrode, as shown in Figure 4c.

### 3.4. Textural Properties

**Figure 5:**
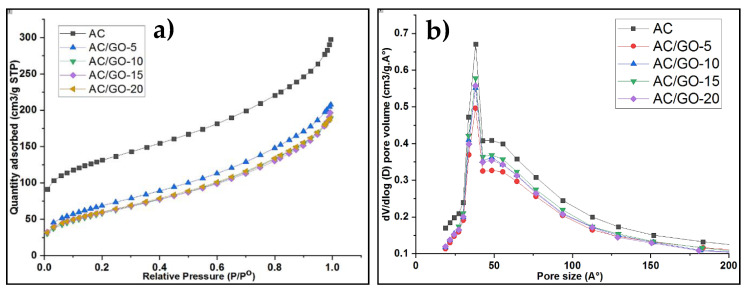
Nitrogen adsorption-desorption isotherm of (**a**) AC and AC/GO-x (**b**) Pore width distribution of AC and AC/GO-x.

In the present work, AC and AC/GO-x (x = 5, 10, 15 and 20 wt.% of GO) electrodes were synthesized and textural properties were compared. As presented in Table 2, there was a sharp decrease in the specific surface area of the activated carbon (AC) powder when converted to an electrode, possibly due to the incorporation of PVDF and there was a further decrease in specific surface area of the composites in comparison to pristine AC electrode, probably due to graphene oxide (GO) addition. The isotherm curves of all the electrode materials possessed a typical type II adsorption isotherm [26], as shown in Figure 5a. Using BJH model, the pore size distribution of our materials was calculated and shown in Figure 5b. Pore size distribution indicates the pores accessible for a molecule or ions of a particular size and shape. Parameters including the total pore volume, BET specific surface area, micropore volume and mesopore volume of AC/GO-*x* are summarized in Table 2.

### 3.5. Wettability Properties

**Figure 6:**
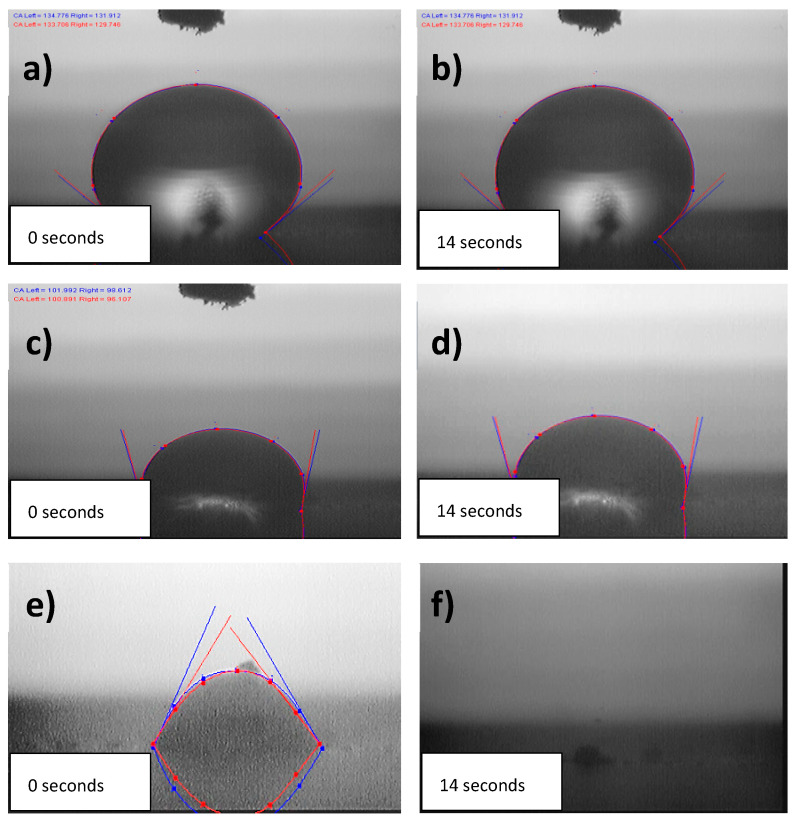
Water Contact Angle of (**a**,**b**) PVDF (**c**,**d**) AC electrode and (**e**,**f**) AC/GO-5 electrode.

The Water Contact Angle (WCA) of the fabricated electrodes (AC and AC/GO-x) was measured to examine their wettability characteristics. Interestingly, all composites have an interesting hydrophilic property over the pristine AC electrode. This is possibly due to the addition of a graphene oxide (GO) that converts the highly hydrophobic PVDF binder (see Figure 6a,b) into a less hydrophobic form that can be seen in Figure 6c–f, hence enabling more penetration of ions into the pores of activated carbon. The addition of PVDF as an additive binder in AC electrode compromised its hydrophilicity; however, when graphene oxide was added to augment this compromise, the hydrophilic nature of the electrode was improved. It is somewhat difficult to capture all the WCA of the composites, as the water dropped on the surface of the materials spread rapidly when in contact with them, hence limited images were captured.

### 3.6. XPS Investigation

**Figure 7:**
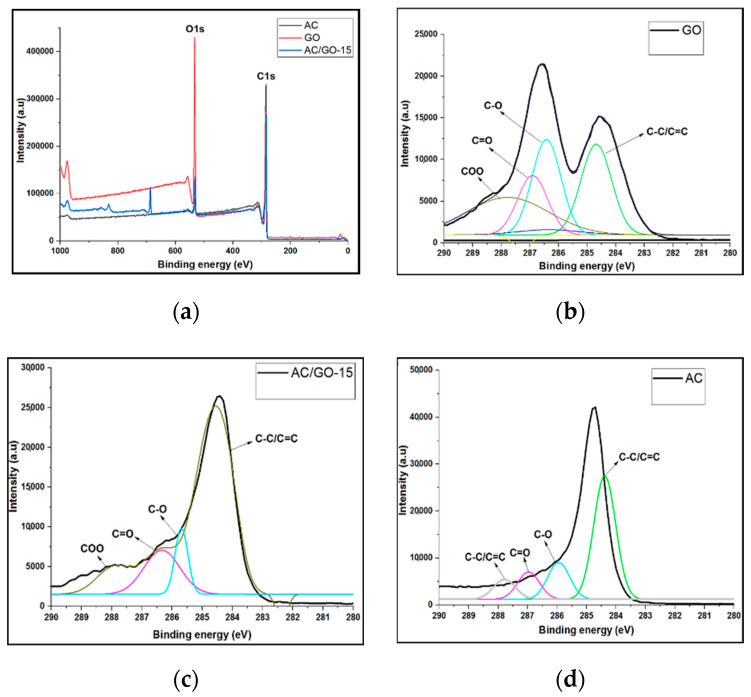
Whole XPS spectra of (**a**) AC, GO and AC/GO-x (where *x* is 15 wt.% GO). XPS spectra of C 1s peak of (**b**) AC (**c**) AC/GO-15 and (**d**) GO.

Further investigation of GO’s influence on the surface chemistry of the composites was revealed by using XPS analysis. For investigation of increased in band of oxygenated functional groups (OFGs), one of the AC/GO-x was chosen and compared with pristine AC and the additive (GO). Figure 7a shows the whole XPS spectra of AC, GO and AC/GO-x. From the spectra, it was obvious that the peak intensity of O element present in AC/GO-x increased upon the addition of GO when compared with pristine AC. The oxygen content of the total element was 5.15% in AC and was 9.51% for AC/GO-x, indicating that some oxygen containing functional groups were added to the composite. The presence of oxygenated functional groups, i.e., (–C=O, COOH etc.) is beneficial for the improvement of electrochemical properties in electric double layer (EDL) capacitors, as the wettability of the capacitor surface is improved [27,28].

Figure 7b–d shows the resolved C 1s spectrum of AC, AC/GO-15 and GO into their individual peaks. Binding energies of 284.9, 286.7 and 288.9 eV present in the distribution of the peaks corresponded to C=C, C-O, C=O and -COO-, respectively.

### 3.7. Electrochemical Properties

**Figure 8:**
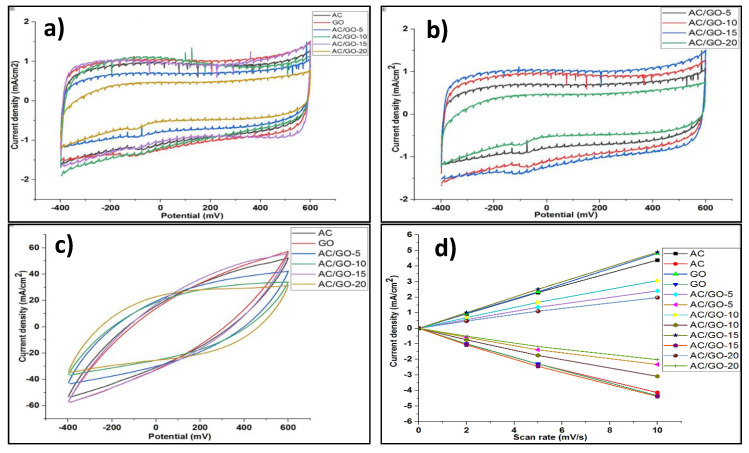
Cyclic voltammetry curve of (**a**) AC, GO and AC/GO-x composite electrodes at the scan rates of 2 mV/s; (**b**) AC/GO-x electrodes at scan rate of 2 mV/s; (**c**) AC, GO and AC/GO-x composite electrodes at the scan rate of 200 mV/s; (**d**) Double-layer capacitance measurements for AC, GO and AC/GO-x composite electrodes voltammetry with 0.5 M NaCl. Cyclic voltammograms were measured in a non-Faradaic region of the voltammogram at low scan rates of 2–10 mV/s. The cathodic (ref current density below 0 mA/cm^2^) and anodic charging currents (ref current density above 0 mA/cm^2^) plotted as a function of scan rate (mV/s). The determined double-layer capacitance of the system was taken as the average of the absolute value of the slope of the linear fits to the data [20].

Cyclic voltammetry (CV) curves of AC and its composite electrodes are shown in Figure 8a–c. At a low scan rate (2 mV/s), an almost rectangular shape was observed in both electrodes, indicating the capacitive nature of the electrodes (stable Electrical Double Layer formation at low scan rate). At a high scan rate of 200 mV/s, (Figure 8c, an oval like pseudo-rectangular shape is observed, which implies a high instability of EDL and poor capacitive nature of the electrodes at high scan rate. Clearly, the AC/GO-15 composite electrode had a higher charging rate and faster ion transport than AC and its counter-part electrodes, possibly due to enhanced hydrophilicity that enables easier surface interaction, thus yielding low ion resistance and better reactivity of the electroactive species caused by the addition of GO at this proportion. The calculated EDL capacitance and specific capacitance of all the electrodes are shown in Table 3.

### 3.8. Desalination Performance

**Figure 9:**
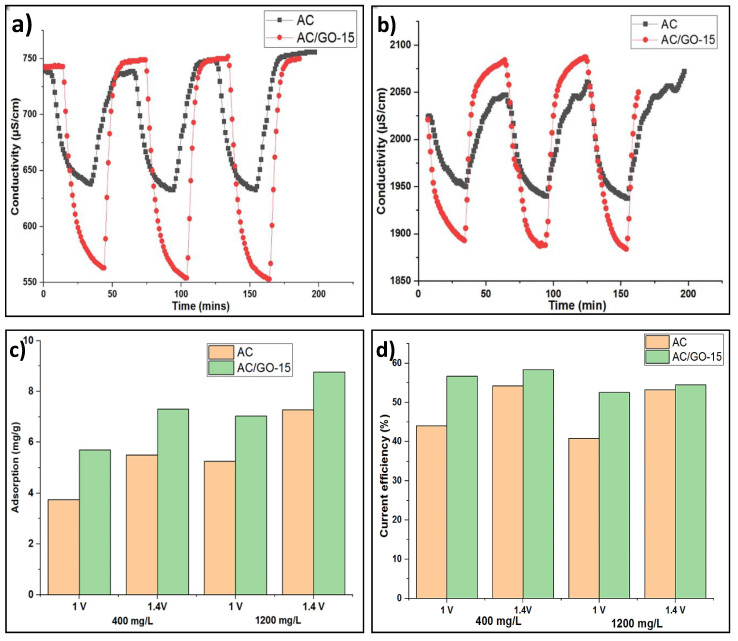
(**a**,**b**) Adsorption curves of AC and AC/GO-15 electrodes at 400 and 1200 mg/L NaCl solution. (**c**) Adsorption behavior of AC and AC/GO-15 electrodes at different NaCl concentrations and operating voltage (**d**) Current efficiency of AC and AC/GO-15 electrodes at different NaCl concentrations and operating voltage.

Based on the outstanding electrochemical properties of AC/GO-15 compared to its counterparts, it was chosen as the electrode of interest, and its propensity for salt adsorption capacity was determined in comparison to pristine AC electrode. An adsorption test was verified by supplying NaCl solution of 400 and 1200 mg/L at a constant flow rate of 25 mL/min after applying a cell potential of 1.0 and 1.4 V for few minutes per cycle to the CDI cell. Three symmetrical cycles of adsorption/desorption were obtained, as shown in Figure 9a,b. The adsorption capacity of the composite AC/GO-15 was measured under the same conditions (400 and 1200 mg/L NaCl at the operating voltage of 1.0 and 1.4 V) with that of the pristine AC electrode.

The introduction of GO into AC undoubtedly improved its hydrophilicity, resulting in an enhanced surface interaction of solvated ions into the pores of the composites, thus yielding a faster rate of salt adsorption (Figure 9c) and a better charge transfer, as shown in Figure 9d. The salt adsorption capacity of AC/GO-15 was 5.70 mg/g while that of the AC electrode was 3.4 mg/g at 400 mg/L NaCl under an operating voltage of 1.0 V. As found in literature, Table 4 gives detailed information of the adsorption capacity of a modified hydrophilic AC electrode/doped GO electrode in CDI operation.

Other CDI operating performance matrices of this experiments under all operating conditions are presented in Table 5. In all cases, the AC/GO-15 showed an overall performance increase over AC.

## 4. Conclusions

In summary, AC and series of AC/GO-x composites (AC/GO-5, AC/GO-10, AC/GO-15 and AC/GO-20) were synthesized and characterized. Among the composite materials, AC/GO-15 possessed the best electrochemical properties and thereafter was utilized as an electrode of interest in comparison with a pristine AC electrode. In all conditions, AC/GO-15 exhibited a higher electro-sorption capacity and better electrochemical performance than an AC electrode. The incorporation of GO in an AC electrode matrix increases the overall hydrophilicity of the material and thus improves the surface interaction of saline solution on the electrode; hence, the consequent effect is made manifest both on the electrochemical and electro-sorption properties of these materials. The electro-sorption capacity of AC/GO-15 was 5.70 mg/g in comparison to 3.74 mg/g of AC electrode. 

From the literature, an improvement in one of the properties of AC affects its electrochemical and adsorption capacity. Advances in improving the hydrophilic nature of AC have been reported but not with any compromise. Acid modification of the surface property of AC leads to attachment of oxygenated functional groups (OFGs,) and this invariably improves its hydrophilicity but could lead to a surface area reduction. Yet, a composite formation of AC with other hydrophilic additives does not provide the coveted overall properties needed, as the additives could block some pores of the AC, thus reducing pores accessibility to ions adsorption. While consideration of additives could be beneficial over acid treatment, both methods have their own shortcomings.

One of the main features of CDI is its material aspect, thus it is necessary to continuously devise means and methods to improve this aspect by using a predictor like a statistical/computer model to dictate a ratio of additives for optimal operation with no significant compromise to the textural properties of AC. This would be a remarkable improvement and can be a focus of research in the future.

## Figures and Tables

**Table 1:** Interlayer spacing with reference to additive.

Sample	d002 (nm)	100/101 (nm)
AC	0.35	0.21
AC/GO-5	0.35	0.21
AC/GO-10	0.35	0.21
AC/GO-15	0.35	0.21
AC/GO-20	0.35	0.21

**Table 2:** Textural parameters of AC, GO and AC/GO-x.

Sample	V_t_ (cm^3^·g^−1^)	S_BET_ (m^2^·g^−1^)	V_MICRO_ (cm^3^·g^−1^)	V_MESO_ (cm^3^·g^−1^)
Pure AC	0.82	1034.02	0.40	0.55
AC	0.33	467.62	0.19	0.35
GO	0.01	5.48	0.02	0.01
AC/GO-5	0.30	228.29	0.07	0.30
AC/GO-10	0.31	231.45	0.18	0.31
AC/GO-15	0.43	240.43	0.08	0.30
AC/GO-20	0.30	200.56	0.06	0.27

**Table 3:** Electrical double layer capacitance (C_EDL_) and specific capacitance (C_Specific_) for AC and AC/GO-x electrodes.

Sample	AC	GO	AC/GO-5	AC/GO-10	AC/GO-15	AC/GO-20
C_EDL_ (F/cm^2^)	0.92	0.58	0.62	0.94	1.61	0.47
Cspecific (F/g)	56	157	43	66	75	33

**Table 4:** Electrosorption behavior of oxidized AC electrode/ doped GO electrode in CDI as found in the literature.

Material	Adsorption Capacity (mg/g)	Adsorption Efficiency (%)	Capacitance (F/g)	Operating Voltage (V)	References
Oxidized AC	-	36.10	8.9	2.00	[29]
“	-	15.00	73.13	“	[30]
“	-	55.00	-	1.00	[31]
“	5.30	-	-	“	[32]
GO/ZrO_2_	4.55	-	-	1.20	[33]
GO/PVA	35.00	-	-	“	[34]
N-doped Ti/GO	9.20	98	157	“	[16]
AC/GO	5.70	20.10	-	1.00	this work

**Table 5:** CDI performance matrices of AC and AC/GO-15 electrodes at all operating conditions.

Electrode	Voltage (V)	Concentration (Mg/L)	Time (min)	Adsorption Efficiency (%)	Salt Adsorption Capacity (SAC, mg/g)	Specific Energy Consumption (SEC) (kWh/m^3^)	Kinetics (mg/min)	Average Salt Adsorption Rate (ASAR mg/g/min)
AC	1.0	400	16	25.14	3.74	0.08	0.56	0.23
AC/GO-15				20.1	5.70	0.10	1.04	0.34
AC	1.4	400	16	37	5.50	0.20	1.2	0.34
AC/GO-15				25.8	7.31	0.18	1.56	0.46
AC	1.0	1200	16	11.76	5.25	0.10	0.55	0.33
AC/GO-15				8.40	7.03	0.09	0.95	0.44
AC	1.4	1200	16	16	7.28	0.26	0.96	0.46
AC/GO-15				10.3	8.77	0.20	1.34	0.55

## References

[1] Alkaisi A., Mossad R., Sharifian-Barforoush A. (2017). A Review of the Water Desalination Systems Integrated with Renewable Energy. Energy Procedia.

[2] Folaranmi G., Bechelany M., Sistat P., Cretin M., Zaviska F. (2020). Towards Electrochemical Water Desalination Techniques: A Review on Capacitive Deionization, Membrane Capacitive Deionization and Flow Capacitive Deionization. Membranes.

[3] Zou L., Li L., Song H., Morris G. (2008). Using mesoporous carbon electrodes for brackish water desalination. Water Res..

[4] Porada S., Zhao R., Van Der Wal A., Presser V., Biesheuvel P.M. (2013). Review on the science and technology of water desalination by capacitive deionization. Prog. Mater. Sci..

[5] Wang X.Q., Wang P., Ning P., Ma Y.X., Wang F., Guo X.L., Lan Y. (2015). Adsorption of gaseous elemental mercury with activated carbon impregnated with ferric chloride. RSC Adv..

[6] Zhang C., Song W., Sun G., Xie L., Wan L., Wang J., Li K. (2014). Synthesis, Characterization, and Evaluation of Activated Carbon Spheres for Removal of Dibenzothiophene from Model Diesel Fuel. Ind. Eng. Chem. Res..

[7] Bonvin F., Jost L., Randin L., Bonvin E., Kohn T. (2016). Super-fine powdered activated carbon (SPAC) for efficient removal of micropollutants from wastewater treatment plant effluent. Water Res..

[8] Wang L., Wang M., Huang Z.-H., Cui T., Gui X., Kang F., Wang K., Wu D. (2011). Capacitive deionization of NaCl solutions using carbon nanotube sponge electrodes. J. Mater. Chem..

[9] Li H., Pan L., Lu T., Zhan Y., Nie C., Sun Z. (2011). A comparative study on electrosorptive behavior of carbon nanotubes and graphene for capacitive deionization. J. Electroanal. Chem..

[10] Ata S., Banerjee S.L., Singha N.K. (2016). Polymer nano-hybrid material based on graphene oxide/POSS via surface initiated atom transfer radical polymerization (SI-ATRP): Its application in specialty hydrogel system. Polymer.

[11] Jia B., Zou L. (2012). Graphene nanosheets reduced by a multi-step process as high-performance electrode material for capacitive deionisation. Carbon.

[12] Kruk M., Jaroniec M. (2001). Gas Adsorption Characterization of Ordered Organic−Inorganic Nanocomposite Materials. Chem. Mater..

[13] Lv J., Zhang G., Zhang H., Yang F. (2018). Graphene oxide-cellulose nanocrystal (GO-CNC) composite functionalized PVDF membrane with improved antifouling performance in MBR: Behavior and mechanism. Chem. Eng. J..

[14] Wu T., Zhou B., Zhu T., Shi J., Xu Z., Hu C., Wang J. (2015). Facile and low-cost approach towards a PVDF ultrafiltration membrane with enhanced hydrophilicity and antifouling performance via graphene oxide/water-bath coagulation. RSC Adv..

[15] Krishnamoorthy K., Navaneethaiyer U., Mohan R., Lee J., Kim S.-J. (2012). Graphene oxide nanostructures modified multifunctional cotton fabrics. Appl. Nanosci..

[16] Khalil K.A., Barakat N.A., Motlak M., Al-Mubaddel F.S. (2020). A novel graphene oxide-based ceramic composite as an efficient electrode for capacitive deionization. Sci. Rep..

[17] Wang Z., Dou B., Zheng L., Zhang G., Liu Z., Hao Z. (2012). Effective desalination by capacitive deionization with functional graphene nanocomposite as novel electrode material. Desalination.

[18] Biswas S., Drzal L.T. (2010). Multilayered Nanoarchitecture of Graphene Nanosheets and Polypyrrole Nanowires for High Performance Supercapacitor Electrodes. Chem. Mater..

[19] Alhabeb M., Beidaghi M., Van Aken K.L., Dyatkin B., Gogotsi Y. (2017). High-density freestanding graphene/carbide-derived carbon film electrodes for electrochemical capacitors. Carbon.

[20] Suryanto B.H., Chen S., Duan J., Zhao C. (2016). Hydrothermally driven transformation of oxygen functional groups at multiwall carbon nanotubes for improved electrocatalytic applications. ACS Appl. Mater. Interfaces..

[21] Dong Q., Wang G., Qian B., Hu C., Wang Y., Qiu J. (2014). Electrospun Composites Made of Reduced Graphene Oxide and Activated Carbon Nanofibers for Capacitive Deionization. Electrochim. Acta.

[22] El-Khodary S.A., El-Enany G.M., El-Okr M., Ibrahim M. (2014). Preparation and Characterization of Microwave Reduced Graphite Oxide for High-Performance Supercapacitors. Electrochim. Acta.

[23] Konwar L.J., Sugano Y., Chutia R.S., Shchukarev A., Mäki-Arvela P., Kataki R., Mikkola J.-P. (2016). Sustainable synthesis of N and P co-doped porous amorphous carbon using oil seed processing wastes. Mater. Lett..

[24] Shen J., Yan B., Shi M., Ma H., Li N., Ye M. (2011). One step hydrothermal synthesis of TiO2-reduced graphene oxide sheets. J. Mater. Chem..

[25] Nishiyama T., Sumihara T., Sato E., Horibe H. (2017). Effect of solvents on the crystal formation of poly(vinylidene fluoride) film prepared by a spin-coating process. Polym. J..

[26] Basri N.H., Dolah B.N.M. (2013). Physical and electrochemical properties of supercapacitor electrodes derived from carbon nanotube and biomass carbon. Int. J. Electrochem. Sci..

[27] Wang K., Zhao N., Lei S., Yan R., Tian X., Wang J., Song Y., Xu D., Guo Q., Liu L. (2015). Promising biomass-based activated carbons derived from willow catkins for high performance supercapacitors. Electrochim. Acta.

[28] Thorogood C.A., Wildgoose G.G., Jones J.H., Compton R.G. (2007). Identifying quinone-like species on the surface of graphitic carbon and multi-walled carbon nanotubes using reactions with 2,4-dinitrophenylhydrazine to provide a voltammetric fingerprint. New J. Chem..

[29] Blegur E.J., Endarko E. (2016). Study the effect of active carbon modified using HNO3 for carbon electrodes in capacitive deionization system. AIP Conf. Proc..

[30] Huang W., Zhang Y., Bao S., Cruz R., Song S. (2014). Desalination by capacitive deionization process using nitric acid-modified activated carbon as the electrodes. Desalination.

[31] Villar I., Roldan S., Ruiz V., Granda M., Blanco C., Menéndez R., Santamariía R. (2010). Capacitive Deionization of NaCl Solutions with Modified Activated Carbon Electrodes S. Energy Fuels.

[32] Gao X., Omosebi A., Landon J., Liu K. (2015). Enhanced Salt Removal in an Inverted Capacitive Deionization Cell Using Amine Modified Microporous Carbon Cathodes. Environ. Sci. Technol..

[33] Yasin A.S., Mohamed H.O., Mohamed I.M., Mousa H.M., Barakat N.A. (2016). Enhanced desalination performance of capacitive deionization using zirconium oxide nanoparticles-doped graphene oxide as a novel and effective electrode. Sep. Purif. Technol..

[34] Leong Z.Y., Lu G., Yang H.Y. (2019). Three-dimensional graphene oxide and polyvinyl alcohol composites as structured activated carbons for capacitive desalination. Desalination.

